# Role of the Ang2–Tie2 Axis in Vascular Damage Driven by High Glucose or Nucleoside Diphosphate Kinase B Deficiency

**DOI:** 10.3390/ijms21103713

**Published:** 2020-05-25

**Authors:** Anupriya Chatterjee, Rachana Eshwaran, Hongpeng Huang, Di Zhao, Martina Schmidt, Thomas Wieland, Yuxi Feng

**Affiliations:** 1 Experimental Pharmacology Mannheim, European Center for Angioscience, Medical Faculty Mannheim, Heidelberg University, 68167 Mannheim, Germany; anupriya.chatterjee@medma.uni-heidelberg.de (A.C.); rachana.eshwaran@medma.uni-heidelberg.de (R.E.); hongpeng.huang@medma.uni-heidelberg.de (H.H.); di.zhao@medma.uni-heidelberg.de (D.Z.); thomas.wieland@medma.uni-heidelberg.de (T.W.); 2 Department of Molecular Pharmacology, University of Groningen, 9713AV Groningen, The Netherlands; m.schmidt@rug.nl; 3 Groningen Research Institute for Asthma and COPD (GRIAC), University Medical Center Groningen, 9700AB Groningen, The Netherlands; 4 DZHK (German Center for Cardiovascular Research), Partner site Heidelberg/Mannheim, 10785 Berlin, Germany

**Keywords:** nucleoside diphosphate kinase B, NDPK-B, angiopoietin 2, Ang2, Tie2, vascular damage, endothelial cells

## Abstract

Ablation of nucleoside diphosphate kinase B (NDPK-B) in mice causes a breakdown of the neurovascular unit in the retina, mimicking diabetic retinopathy. The NDPK-B deficiency-induced vascular damage is mediated by excessive angiopoietin 2 (Ang2). Herein, the potential involvement of its receptor, Tie2, was investigated. NDPK-B-deficient mouse retinas showed an upregulation of Tie2, specifically in the deep capillary layer. A similar upregulation of Tie2 was observed in cultured endothelial cells (ECs) from different origins upon NDPK-B depletion, whereas high glucose (HG) treatment did not alter Tie2 expression. Immunofluorescence staining and subcellular fractionation showed that the majority of Tie2 upregulation occurred at the plasma membrane. Similar to HG, however, NDPK-B depletion reduced Tie2 tyrosine phosphorylation. Compared to HG, a stronger increase of Ang2 was observed in NDPK-B depleted ECs. Treatment of ECs with soluble Tie2 or siRNA-mediated Tie2 knockdown attenuated NDPK-B depletion- but not HG-induced Ang2 upregulation. Like NDPK-B depletion, overexpression of recombinant Ang2 in ECs enhanced Ang2 secretion and concomitantly promoted the upregulation of Tie2. Thus, we identified a new mechanism showing that after reaching a threshold level of secretion, Ang2 sustains its own expression and secretion by a Tie2-dependent positive feedback loop.

## 1. Introduction

Nucleoside diphosphate kinases (NDPKs) are housekeeping enzymes primarily involved in nucleoside triphosphate homeostasis in the cell [1]. Besides this canonical function, an increasing number of publications show additional functions and highlight the moonlighting character of several isoforms [2,3,4]. The isoform B (NDPK-B) acts as a protein histidine kinase on several targets such as the G protein β subunit and the ion channels KCa3.1 and TRPV5 [3,5].

Dysregulated permeability is a hallmark of vascular disorders that affect blood barriers of the brain and retina, an extension of the brain [6,7]. Interestingly, NDPK-B apparently contributes to endothelial barrier function. Thus, NDPK-B-deficient mice exhibit increased vascular permeability in the brain compared to their wild type counterparts [8]. Seemingly, NDPK-B controls vascular permeability by regulating the distribution of caveolins and adherens junction proteins in endothelial cells (ECs), thereby contributing to pathological angiogenesis [8,9]. Recently, we discovered that NDPK-B^−/−^ mice develop a breakdown of the neurovascular unit (NVU) in the retina, mimicking diabetic retinopathy (DR) without existing hyperglycemia. The NVU consists of endothelial cells (ECs), pericytes (PC), glial cells, and neurons. The cellular interaction between these cells in the retina enables the maintenance of proper retinal functions. Emerging evidence demonstrates that multiple factors contribute to retinal homeostasis. NDPK-B depletion in these retinas results in a loss of pericytes and an increase in acellular capillaries (AC). In accordance with DR, the elevation of the vascular growth factor angiopoietin 2 (Ang2), has been identified as a driving force for the pathology [10]. Therefore, the hyperglycemia- as well as the NDPK-B deficiency-induced vascular damage was prevented in mice haplodeficient for the Ang2 encoding Angpt2 gene [11].

ECs are the major source of the Ang2 increase in hyperglycemic as well as NDPK-B-deficient retinas [11,12]. Tie2, the receptor of Ang2, is predominantly expressed in ECs and regulates endothelial cell survival, proliferation, and migration, implicating vascular integrity, angiogenesis, and/or vascular regression [13,14,15]. Angiopoietin 1 (Ang1), the canonical agonist of Tie2, is released by perivascular cells and binds to Tie2 to promote endothelial survival, adhesion, and quiescence [16]. Upon binding of Ang1, Tie2 is phosphorylated, which further activates survival signaling via phosphorylation of PI3K, Akt, and FoxO1 [17]. The binding of Ang2 to Tie2, however, has context-dependent outcomes [18]. As a known antagonist, it perturbs Ang1/Tie2 signaling and promotes vascular remodeling via the loosening of matrix contacts. It supports the effects of the vascular endothelium-derived growth factor (VEGF) in inducing angiogenic responses. In the absence of or at low VEGF levels, however, excessive Ang2 leads to vascular regression [19]. Nevertheless, several reports indicate that Ang2 might be a weak agonist of Tie2, promoting its phosphorylation and vascular survival [20,21]. It has been proposed that hyperglycemia increases intracellular methylglyoxal which, through modification of mSin3a, activates Ang2 transcription [22]. However, a potential contribution of Tie2 in Ang2-driven vascular damage in NDPK-B-deficient conditions has not been studied.

In this study, we therefore investigated the role of the Tie2 receptor in NDPK-B-deficient retinas and ECs by manipulating the Ang2–Tie2 signaling axis upon high glucose (HG) treatment or NDPK-B depletion. We provide evidence that exorbitant Ang2 stimulates its own expression and secretion via a Tie2-dependent positive feedback loop. Our data clarify the signaling pathways that enhance Ang2 expression and secretion and thus promote vascular damage in the retina.

## 2. Results

### 2.1. Tie2 Is Upregulated in the NDPK-B-Deficient Retina

**Figure 1:**
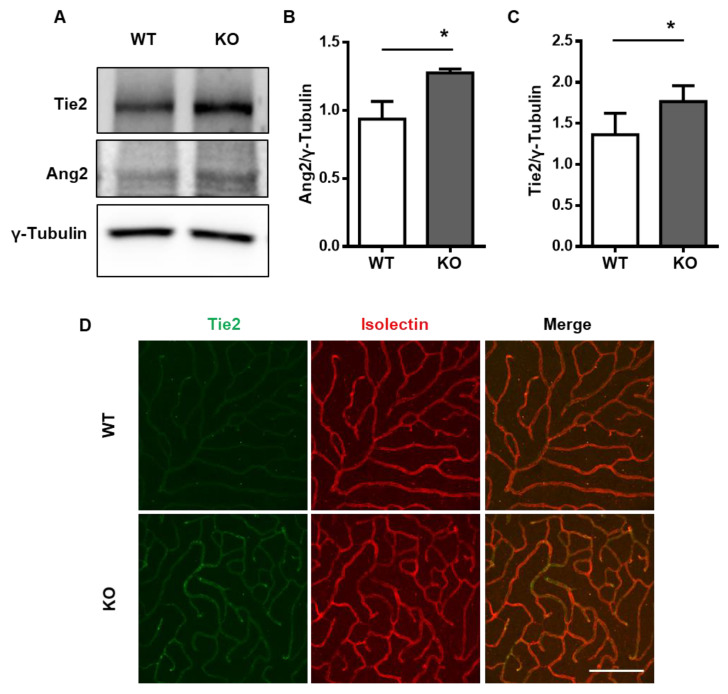
Tie2 is upregulated in the NDPK-B-deficient retina. (**A**) Representative immunoblots of Tie2, Ang2, and γ-Tubulin in NDPK-B^−/−^ retinal lysates. (**B**,**C**) Quantification of Ang2 and Tie2, respectively, normalized to γ-tubulin, showing upregulation of Tie2 in NDPK-B-deficient retinas (*n* = 5), WT: wild type, KO: NDPK-B^−/−^, * *p* < 0.05. (**D**) Expression of Tie2 (green) and Lectin (red) in the deep capillary layer of wild type (WT) and NDPK-B^−/−^ (KO) retinas, depicting elevated Tie2 in the deep capillary layer of NDPK-B^−/−^ retina compared with control retina. The pictures shown are representative of staining from three animals in each group, scale bar 50 µm.

To investigate the Ang2–Tie2 axis in the retina under NDPK-B-deficient conditions, we first assessed the expression of Tie2 in 5-month-old NDPK-B^−/−^ (KO) mouse retinas by immunoblotting. Similar to prior findings, Ang2 was significantly upregulated in KO retinas compared to wild type (WT) by about 30% (Figure 1A,B). Similarly, the level of Tie2 in the KO retinas was also about 30% higher than in the WT retinas (Figure 1A,C). To identify the cellular localization of Tie2 upregulation in the NDPK-B KO retinas, we further examined the expression of Tie2 in the retina via whole-mount immunofluorescence staining. Isolectin staining distinguished the vasculature from the surrounding retinal tissue (Figure 1D). Tie2 was detected in the entire retinal vasculature. Its expression was unaltered between the KO and WT arterioles, venules, as well as the superficial capillary layer. However, in the deep capillary layer, where pathological vascular degeneration commonly starts, higher Tie2 expression was observed in the KO retinas compared to the WT (Figure 1D). Taken together, the data show a concomitant Tie2 upregulation in the KO retinas, especially in the deep capillary layer, hinting towards a potential contribution of Tie2 as a mediator of the vasoregressive phenotype.

NDPK-B KO retinas largely mimic diabetic retinas regarding the upregulation of Ang2 and the subsequent development of vascular degeneration [10]. To determine whether Tie2 expression in the diabetic retina resembles the NDPK-B KO retina, 3-month diabetic mouse retinal lysates were assessed by immunoblotting. Since the Tie2 expression in the retina was not altered in the diabetic retinas, the phenotype of NDPK-B KO retinas apparently differs from that of diabetic retinas with regard to Tie2 expression levels.

### 2.2. NDPK-B Depletion Upregulates Ang2 and Tie2 in Micro- and Macrovascular Endothelial Cells

**Figure 2:**
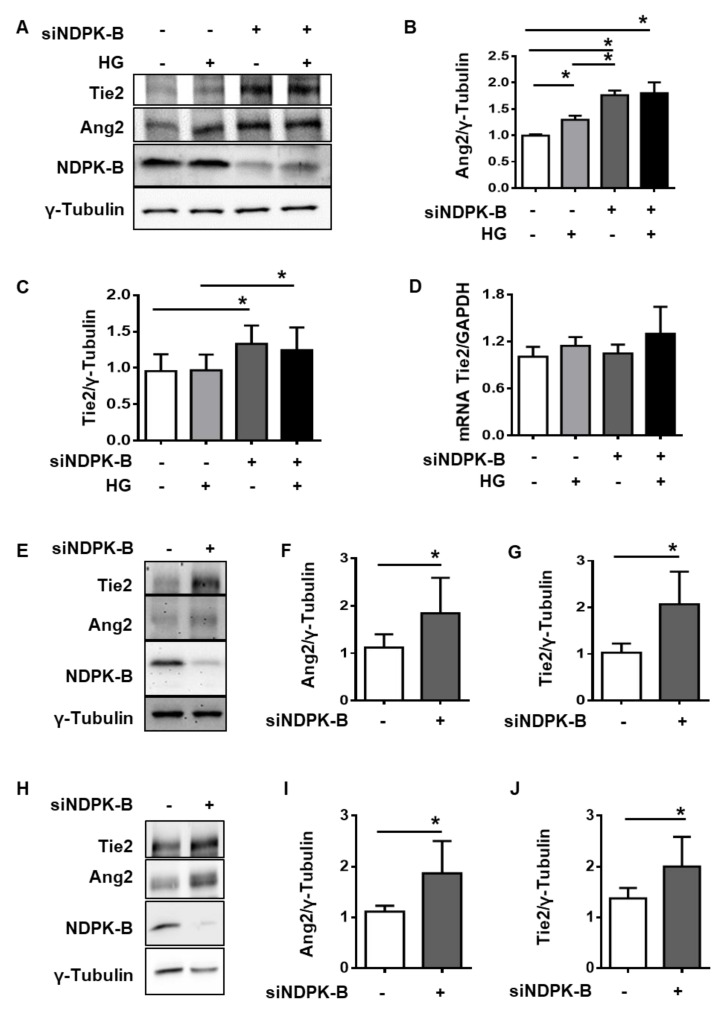
NDPK-B depletion upregulates Ang2 and Tie2 in endothelial cells. (**A**) HUVECs were transfected with either scrambled control (−) or siRNA against NDPK-B (siNDPK-B, +). (**A**) Representative immunoblots of Tie2, Ang2, NDPK-B, and γ-Tubulin in NDPK-B depleted HUVECs treated for 24 h without (−) or with HG, (+). Quantification of Ang2 (**B**) and Tie2 (**C**) levels, (*n* = 4). (**D**) Tie2 mRNA expression normalized to glyceraldehyde 3-phosphate dehydrogenase (GAPDH) mRNA (*n* = 4). HMECs and HRMVECs were transfected with either scrambled control (−) or siRNA against NDPK-B (siNDPK-B, +). (**E**) Representative immunoblots of Tie2, Ang2, NDPK-B, and γ-Tubulin in HMECs. Quantification of Ang2 (**F**) and Tie2 (**G**) levels, (*n* = 3). (**H**) Representative immunoblots of Tie2, Ang2, NDPK-B, and γ-Tubulin in HRMVECs. (**I**,**J**) Quantification of Ang2 (**I**) and Tie2 (**J**) levels, (*n* = 5). All protein contents were normalized to γ-Tubulin. * *p* < 0.05.

To confirm the endothelial regulation of Tie2 detected in the retina and further analyze the role of the Ang2–Tie2 axis upon loss of NDPK-B and hyperglycemia, we cultured macrovascular human umbilical vein endothelial cells (HUVECs), in which the efficient siRNA-mediated knockdown of NDPK-B was already established (Figure 2A, [10]), in medium containing normal (5 mM) or high glucose (30 mM, HG) for 24 h. In accordance with the published data, the upregulation of Ang2 was observed in HG as well as NDPK-B depleted ECs (Figure 2A,B). The increase in Ang2 levels was, however, significantly (about 2-fold) more pronounced in NDPK-B-depleted than in HG-treated ECs. As reported before [10], the combination of NDPK-B depletion and HG treatment did not further increase Ang2 levels. In accordance with the data obtained from NDPK-B-deficient and diabetic retinas, Tie2 was significantly elevated upon NDPK-B knockdown but remained unaltered upon HG treatment (Figure 2A,C).

To determine whether Tie2 upregulation upon NDPK-B knockdown occurs due to transcriptional regulation, we analyzed its mRNA expression. Neither NDPK-B depletion nor HG treatment altered Tie2 mRNA content (Figure 2D). Taken together, these findings indicate that an enhanced Ang2 expression, as occurs, for example, upon NDPK-B knockdown in HUVECs, is associated with an increase in endothelial Tie2 levels.

In order to verify that the increase in Tie2 levels is dependent on NDPK-B depletion and a common regulation in ECs, two microvascular endothelial cell lines were analyzed. Human microvascular endothelial cells (HMECs) are commercially available immortalized microvascular ECs of dermal origin. Like in HUVECs, NDPK-B was efficiently depleted in HMECs via siRNA-mediated knockdown. Ang2 levels were increased by 1.75-fold in NDPK-B depleted HMECs (Figure 2E,F). Concomitant with the Ang2 elevation, Tie2 expression increased by about 2-fold (Figure 2E,G). To further corroborate these findings, NDPK-B was also depleted in human retinal microvascular endothelial cells (HRMVECs). Unlike HMECs, HRMVECs are a non-immortalized primary cell population isolated from the retina of a single donor. In HRMVECS as well, NDPK-B depletion induced a significant elevation of Ang2 and Tie2 as compared to the control group (Figure 2H–J).

### 2.3. NDPK-B Depletion Enhances Tie2 Levels at the Cell Membrane

**Figure 3:**
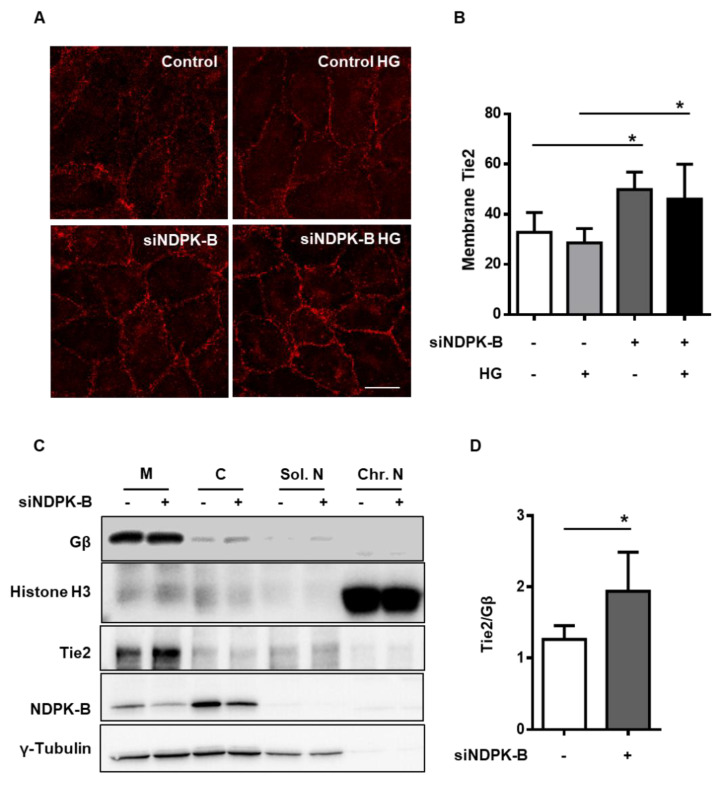
NDPK-B depletion enhances Tie2 content at the cell membrane. HUVECs were transfected with either scrambled control (−) or siRNA against NDPK-B (siNDPK-B, +) and treated for 24 h without (−) or with HG (+) if indicated. (**A**) Representative immunofluorescence images of Tie2 expression in NDPK-B depleted ECs with or without HG treatment. (**B**) Quantification of membrane Tie2 as pixel density in (**A**). (**C**) Membrane (M), Cytosolic (C), soluble nuclear (Sol. N), and chromatin nuclear (Chr. N) fractions were prepared from control and NDPK-B depleted cells. Representative immunoblots showing the subcellular localization of Tie2 in control and NDPK-B depleted ECs. (**D**) Tie2 was quantified relative to the membrane-anchored protein Gβ (*n* = 4). * *p* < 0.05. Scale bar 20 µm.

Subsequently, we investigated the localization of endothelial Tie2. Immunofluorescence staining showed that Tie2 was localized at the cell membrane as well as in intracellular compartments (Figure 3A). HG treatment did not noticeably alter the subcellular localization of Tie2. After NDPK-B knockdown, the overall Tie2 content seemed to be elevated throughout the EC but especially appeared to be increased at the plasma membrane. The quantification of Tie2 levels showed a significant increase at the plasma membrane by about 1.5-fold upon NDPK-B knockdown, but no alteration was detected upon HG treatment (Figure 3B).

To substantiate these findings, we used subcellular fractionation to analyze the Tie2 content in different cellular compartments of control and NDPK-B-depleted ECs by immunoblotting. As shown in Figure 3C, the identity of the membrane fraction was validated by Gβ, and the chromatin containing nuclear fraction by Histone H3. As expected, γ-Tubulin was present in the membrane, cytoplasmic, and soluble nuclear fractions. NDPK-B was detected largely in the cytoplasmic fraction and partially at the membrane. Its knockdown reduced its expression in both these cellular compartments. Tie2 was predominantly detected at the membrane and to a much lesser extent in the other fractions. Tie2 was also detected in the nuclear fraction. In accordance with the immunostainings, the membrane content of Tie2 was increased about 1.8-fold in NDPK-B-depleted cells (Figure 3D), suggesting that NDPK-B knockdown caused an enrichment of Tie2 at the plasma membrane.

### 2.4. NDPK-B Depletion and High Glucose Reduce Tie2 Phosphorylation due to Elevated Ang2 Secretion

**Figure 4:**
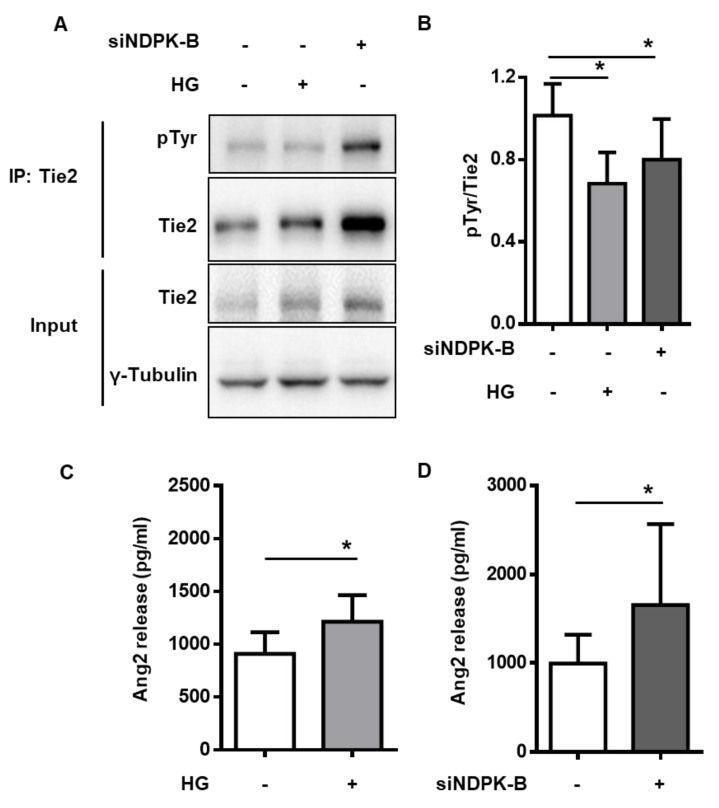
NDPK-B depletion and HG treatment reduce Tie2 phosphorylation due to elevated Ang2 secretion. HUVECs were transfected with either scrambled control (−) or siRNA against NDPK-B (siNDPK-B, +) or treated for 15 min without (−) or with HG (+) if indicated. (**A**) Representative immunoblots of tyrosine phosphorylation (pTyr) of Tie2 after immunoprecipitation (IP); Tie2 and γ-Tubulin content in whole-cell lysates (Input) are also shown. (**B**) Quantification of Tie2 phosphorylation relative to the total amount of precipitated Tie2 (*n* = 3). HUVECs were transfected with either scrambled control (−) or siRNA against NDPK-B (siNDPK-B, +) or treated for 24 h without (−) or with HG) (+). Quantification of secreted Ang2 in the supernatant of HG-treated (**C**, *n* = 7) and NDPK-B depleted ECs (**D**, *n* = 5) by ELISA * *p* < 0.05.

At the plasma membrane, Tie2 functions as a receptor for angiopoietins. Its activity can be monitored by its autophosphorylation on tyrosine residues [19,21]. Therefore, Tie2 was immunoprecipitated and its autophosphorylation was detected by immunoblot with a phospho-tyrosine (pTyr) specific antibody (Figure 4A). Also upon immunoprecipitation, an increase in total Tie2 levels after NDPK-B depletion was detected, whereas no significant change was caused by HG treatment. Similarly, the total phosphorylation of Tie2 was elevated after NDPK-B depletion. However, the relative Tie2 phosphorylation (pTyr/Tie2), which monitors the proportion of activated receptors, revealed an approximate 40% decrease upon HG treatment and a 25% decrease after NDPK-B depletion (Figure 4A,B). These data indicate a relative decrease in the activation of Tie2 receptors despite the higher amount of membranous Tie2 after the loss of NDPK-B. As Ang2 is generally believed to act as an antagonist of Tie2, such a reduction could be caused by an enhanced Ang2 secretion by ECs. As a mediator of vascular remodeling, endothelial Ang2 is stored in Weibel-Palade bodies and secreted rapidly upon stimulation [23].

To investigate whether the increase in the cellular content of Ang2 levels is reflected in its secretion, we quantified Ang2 in the supernatants of cultured ECs by enzyme-linked immunosorbent assay (ELISA). HG treatment caused a 1.25-fold increase in Ang2 secretion compared to normal glucose treated controls (Figure 4C). Similarly, NDPK-B knockdown induced an elevation in Ang2 secretion. In accordance with the data on the increase of total Ang2 levels in NDPK-B depleted cells (see Figure 2), the secreted Ang2 levels were also about 1.6-fold higher than in the supernatant of control transfected cells (Figure 4D). The amount of Ang2 secreted from NDPK-B depleted ECs was approximately 300 pg/mL higher than in HG-stimulated ECs.

### 2.5. Tie2 Is Required for NDPK-B Depletion-Induced Ang2 Upregulation

**Figure 5:**
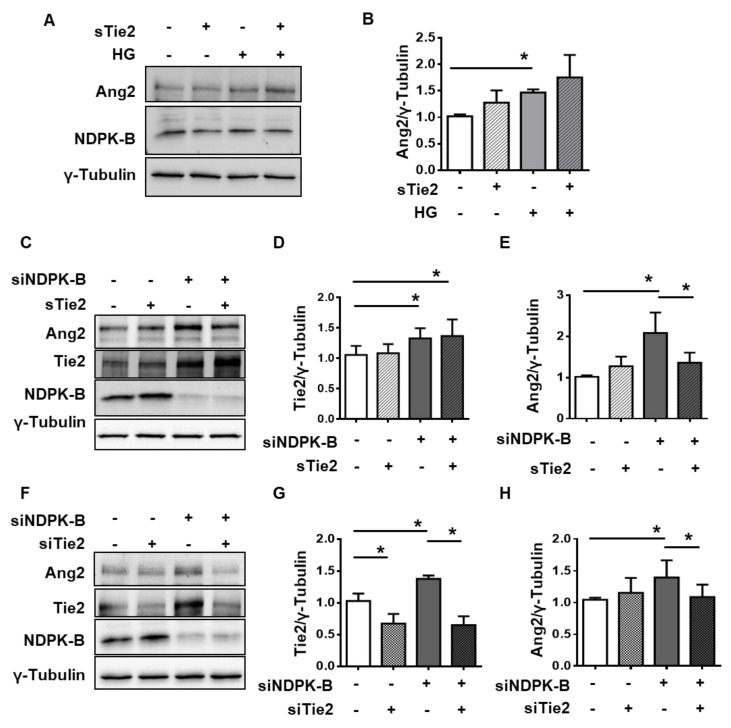
Tie2 is required for NDPK-B depletion- but not HG-induced Ang2 upregulation. HUVECs were transfected with either scrambled control (−) or siRNA against NDPK-B (siNDPK-B, +), treated without (−) or with HG (+) and without (−) or with soluble Tie2 (sTie2, +) for 24 h. (**A**,**C**) Representative immunoblots for Ang2, NDPK-B, Tie2, and γ-Tubulin as indicated. (**B**) Quantification of Ang2 normalized to γ-Tubulin in HG and sTie2 treated cells (*n* = 4). Quantification of Ang2 (**D**) and Tie2 (**E**) normalized to γ-Tubulin in NDPK-B-depleted and sTie2 treated cells, (*n* = 4). HUVECs were transfected with either scrambled control (−) or siRNA against NDPK-B (siNDPK-B, +) or Tie2 (siTie2, +). (**F**) Representative immunoblots for Ang2, Tie2, NDPK-B, and γ-Tubulin in cells depleted of NDPK-B and Tie2. Quantification of Tie2 (**G**) and Ang2 (**H**) normalized to γ-Tubulin (*n* = 4). * *p* < 0.05.

In order to examine the importance of this increase in extracellular Ang2 levels for Tie2 receptor function in NDPK-B depleted ECs, we interrupted the binding of Ang2 to membranous Tie2 by adding soluble Tie2Fc (sTie2), the naturally occurring ectodomain of the Tie2 receptor. It acts as a scavenger of extracellular angiopoietins, thus blocking the phosphorylation and activation of membranous Tie2 [24]. The addition of sTie2 had no effect on Ang2 levels in control ECs (Figure 5A). As shown before, HG treatment induced a 1.5-fold increase in Ang2 expression (Figure 5A,B). NDPK-B-depleted and control ECs were also treated with sTie2 (Figure 5C,D). Tie2 expression was again elevated in the knockdown ECs but not altered in the presence of sTie2 (Figure 5C,D). Interestingly, sTie2 altered the upregulation of Ang2 upon NDPK-B depletion. Whereas NDPK-B knockdown induced a 2-fold increase in Ang2 levels (Figure 5C,E), this increase was abrogated in the presence of sTie2. These data imply that membrane ligand-Tie2 interaction contributes to Ang2 upregulation upon NDPK-B depletion.

To verify the requirement of Tie2 for NDPK-B depletion-induced Ang2 upregulation, we also reduced the Tie2 expression level in ECs via siRNA-mediated knockdown. The total Tie2 content was diminished significantly by about 40% upon knockdown (Figure 5F,G). In Tie2-NDPK-B double depleted cells, an elevation of Tie2 was not detectable (Figure 5F,G). Although the Tie2 depletion had no effect on the basal levels of Ang2, it effectively suppressed the NDPK-B knockdown-induced elevation of Ang2 (Figure 5F,H). Taken together, the data indicate that the activity of membranous Tie2 contributes to the upregulation of Ang2 in NDPK-B depleted ECs.

### 2.6. Excess Secreted Ang2 Promotes Upregulation of Tie2

**Figure 6:**
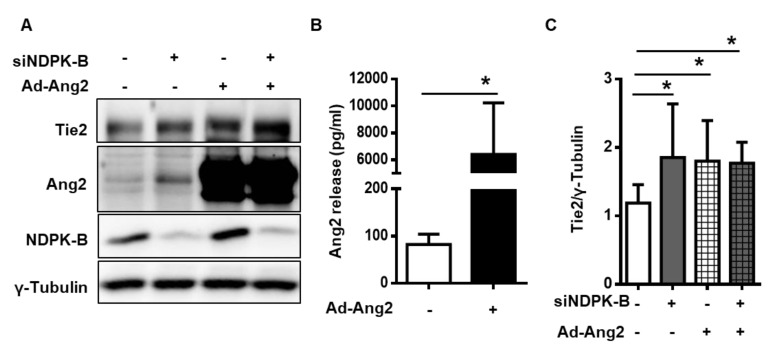
An excess of secreted Ang2 promotes the upregulation of Tie2. HUVECs were transfected with either scrambled control (−) or siRNA against NDPK-B (siNDPK-B, +) and infected with an Ang2 encoding adenovirus (Ad-Ang2, +) or Ad-GFP (−) as control. (**A**) Representative immunoblots of Tie2, Ang2, NDPK-B, and γ-Tubulin in NDPK-B depleted cells with and without Ang2 overexpression. (**B**) Quantification of secreted Ang2 in cells without and with Ang2 overexpression (Ad-Ang2, *n* = 4). (**C**) Quantification of Tie2 normalized to γ-Tubulin (*n* = 6). * *p* < 0.05.

As proof of concept that the upregulation of Tie2 upon NDPK-B depletion occurs in response to an increase in Ang2 secretion, we mimicked this condition by adenoviral overexpression. As shown in Figure 6A, Ang2 was robustly overexpressed 24-h post-infection. The secretion of Ang2 as detected by ELISA confirmed markedly higher Ang2 levels in the supernatant of infected ECs (Figure 6B). Interestingly, Tie2 was elevated to a similar extent in ECs overexpressing Ang2 as in those with NDPK-B depletion (Figure 6A,C). Collectively, our findings prove that upon NDPK-B depletion, and Tie2 upregulation is mediated by increasing extracellular levels of Ang2.

## 3. Discussion

In this study, we demonstrated that concomitant with a stronger upregulation of Ang2, NDPK-B deficiency but not hyperglycemia increases retinal and endothelial Tie2 levels. We identified a novel mechanism involved in Ang2 regulation showing that excessive elevated Ang2 in NDPK-B deficiency compared to high glucose conditions triggers an increase of the Tie2 content in the plasma membrane of endothelial cells. This increase in Tie2 further supports Ang2 upregulation and release in a positive feedback loop, thereby likely contributing to the progression of the NVU breakdown.

Herein we report for the first time a positive feedback loop via the Ang2–Tie2 interaction in NVU injury. Enhanced Ang2 production and secretion has already been linked to the initiation and development of vascular degeneration in the diabetic retina. Rangasamy et al. demonstrated that Ang2 released by ECs regulates endothelial VE-cadherin distribution by altering the phosphorylation of VE-cadherin, leading to the breakdown of the NVU [25]. Our own previous data showed an upregulation of Ang2 in NDPK-B-deficient retinas displaying a vasoregressive phenotype [10]. As shown here, the increase in Ang2 levels was significantly more pronounced upon NDPK-B depletion when compared to high glucose treatment, highlighting again the importance of Ang2 in the regulation of the NVU, but indicating diversity in the pathways regulating Ang2.

We further addressed the potential role of the Ang2–Tie2 axis in NDPK-B deficiency-mediated Ang2 upregulation. In accordance with previous studies [26], Tie2 expression was unaltered by hyperglycemia in diabetic retinas. In contrast, Tie2 was upregulated in NDPK-B-deficient retinas, predominantly in the retinal deep capillary layer where vascular degeneration preferentially initiates. We further demonstrated that the upregulation of Tie2 caused by the loss of NDPK-B is not confined to the microvasculature, but occurs also in diverse vascular beds. Notably, the increase in Tie2 levels occurred at the plasma membrane and was not reflected by an increase in Tie2 gene transcription.

Previously, we showed that NDPK-B depletion in ECs interferes with caveolin-1 transport and caveolae formation. Therefore, an increase in the membrane content of Tie2, as detected herein, might be a result of its stabilization, preventing its internalization and degradation. Indeed, Tie2 colocalizes with caveolin-1 in the caveolae of ECs [27], and Tie2 internalization in the presence of excess Ang2 is unchanged.

Tie2 is known to regulate signaling in a context- and phosphorylation-dependent manner due to the fact that Ang2 exhibits antagonistic and agonistic actions on Tie2 [21,28,29]. On the one hand, Ang2 inhibits the Ang1-mediated survival-promoting activation of Tie2 in pathological conditions. On the other hand, in non-pathological conditions, it can act as an agonist itself, promoting Tie2 phosphorylation [21]. Our findings support reports by others showing a decrease in Tie2 phosphorylation by hyperglycemia as a measure of its activity [26,30]. This suppression of the phosphorylation of Tie2 likely occurs via the inhibitory action of the secreted Ang2 on Tie2. In line with this interpretation, the fraction of phosphorylated Tie2 was also reduced upon NDPK-B depletion, although the total amount of Tie2 was increased. Inhibition of Tie2 phosphorylation detected in NDPK-B depleted ECs implies an antagonistic effect similar to those in studies reported in pathological conditions such as diabetic retinopathy.

**Figure 7:**
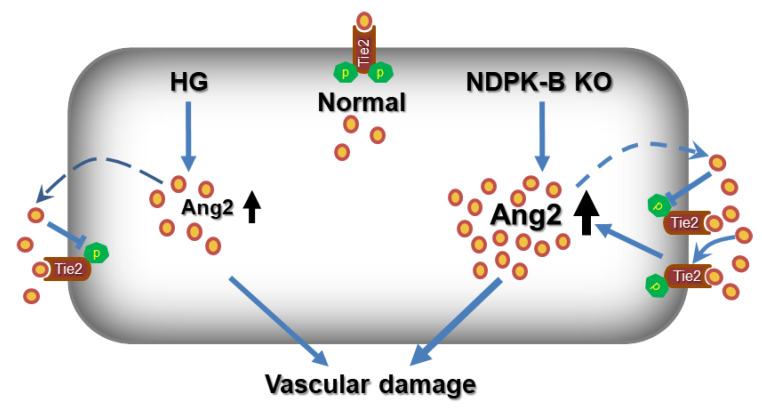
Model of the differential regulation of Ang2 via Tie2 in NDPK-B deficiency- and hyperglycemia (HG)-induced vascular regression. HG moderately upregulates the expression and secretion of Ang2, which contributes to vascular regression, via diminished Tie2 signaling. Similarly, NDPK-B deficiency induces upregulation and secretion of Ang2, which is also associated with a diminished proportion of activated Tie2. Under these conditions, however, the Ang2 levels reach a threshold in which the total amount of Tie2 is additionally increased. By its interaction with Tie2, Ang2 increases its own production and secretion in a positive feedback loop which might be an important driving force in NDPK-B deficiency-induced vascular regression.

In addition to the regulation of Tie2 by phosphorylation, its overexpression has been demonstrated in vessels of tumors, psoriasis, and pulmonary hypertension. However, the exact role of Tie2 signaling in these diseases is not yet elucidated. Based on the data showing increased Ang2 secretion upon its overexpression, as well as the higher Ang2 secretion in NDPK-B depleted ECs, we hypothesize that after reaching a threshold, Ang2 might foster its own expression and secretion from ECs in a manner dependent on the Tie2 level in the plasma membrane (Figure 7). This hypothesis is supported by the results of the experiments using sTie2 and siTie2 to interrupt Tie2 signaling. As Ang1 is secreted from periendothelial cells, not from ECs, it is absent in cultured ECs. Thus, we can use sTie2 to address the role of secreted Ang2 upon NDPK-B depletion. Interestingly, by sequestering secreted Ang2, the relatively strong increase in the amount of cellular Ang2 upon NDPK-B depletion was prevented, whereas the smaller increase in Ang2 levels upon HG treatment was not. Although Ang2 is able to interact with other receptors on endothelial cells, such as integrins to modulate vascular remodeling [31,32,33], the data obtained upon Tie2 depletion point to the Ang2–Tie2 interaction as a requirement for such a feed-forward loop.

Nevertheless, Daly et al. showed that the activation of FoxO1 by overexpressing the phosphorylation mutant to AKT promoted endogenous Ang2 expression and release in ECs. Decreased Tie2 expression and increased Tie2 phosphorylation were correlated with the increase in endogenous Ang2 levels [28]. However, we observed contrary results showing increased Tie2 expression and decreased Tie2 phosphorylation in NDPK-B-deficient conditions. Apparent discrepancies between the studies may be explained by different study designs. The regulation of Tie2 in their study is mediated by FoxO1-induced Ang2 mRNA transcriptional activation, whereas Ang2 mRNA is not altered in NDPK-B-deficient conditions. Moreover, our publication demonstrated that NDPK-B deficiency-induced Ang2 regulation is mediated by FoxO1 via protein O-GlcNAcylation instead of phosphorylation, indicating multiple regulation possibilities contributing to the Ang2–Tie2 signaling pathway [11].

Taken together, our study outlines a novel modulation of the Ang2–Tie2 axis in endothelial cells, which is induced by NDPK-B deficiency but not by hyperglycemia/HG (Figure 7). Although both NDPK-B deficiency and HG induced upregulation of Ang2, suppressing Tie2 phosphorylation, only NDPK-B deficiency provoked a positive feedback loop in which Tie2 is overexpressed, inducing an enhancement of Ang2 that further promotes its elevation, and is in accordance with the important role of Ang2 in NDPK-B deficiency-driven endothelial damage [10,11]. Our findings identify Tie2 as a key regulator in NDPK-B deficiency-driven endothelial damage. Several publications explore the possibility of targeting Tie2 in vascular disorders during NVU breakdown, featuring high circulating Ang2 for therapeutic intervention [34,35]. Therefore, our data might be of relevance for several vascular diseases and provide novel insights into the mechanisms of Ang2–Tie2 signaling in ECs.

## 4. Materials and Methods

### 4.1. Materials and Reagents

soluble Tie2-Fc chimera human (sTie2) (C-68028, Promokine, Germany, 5 µg/mL), D-Glucose (G7021, Sigma, Germany 5 mM or 30 mM), gelatin from porcine skin (48720, Fluka, Bucharest, Romania, 1% solution in PBS) was used to coat cell culture and assay plates. The antibodies used were: mouse anti-NDPK-B (Kamiya Biomedicals, Seattle, WA, USA; MC-412); mouse anti-Ang2 (sc-74403, Santa Cruz Biotechnology, Dallas, TX, USA); rabbit anti-Tie2 (Santa Cruz Biotechnology, Dallas, TX, USA, sc-324); mouse anti-pTyrosine 4G10 (Millipore, Burlington, MA, USA; 05-321); mouse anti-γ-tubulin (Sigma-Aldrich, St. Louis, MO, USA; T6557); rabbit anti-Gβ (Santa Cruz Biotechnology, Dallas, TX, USA; sc-378); goat anti-Histone H3 (Santa Cruz Biotechnology, Dallas, TX, USA; sc-8654); Isolectin-TRITC (Sigma-Aldrich, St. Louis, MO, USA; L5264); goat anti-Tie2 (R&D Systems, Minneapolis, MN, USA; AF762); mouse anti-Tie2/TEK clone AB33 (Merck, Darmstadt, Germany; 05-584) rabbit anti-goat peroxidase (Sigma-Aldrich, St. Louis, MO, USA; A8919); rabbit anti-mouse peroxidase (Sigma-Aldrich, St. Louis, MO, USA; A9044); goat anti-rabbit peroxidase (Sigma-Aldrich, St. Louis, MO, USA; A9169); donkey anti-goat-FITC (Acris Antibodies, Hiddehausen, Germany; R1254F); swine anti-rabbit-FITC (Dako Agilent Technologies, Santa Clara, CA, USA; F0205)

### 4.2. Animals

The use of mice in the study was approved by the ethics committees Regierungspräsidium Karlsruhe (35-9185.81/G-178/15, 2015). The care and experimental use of animals were in accordance with institutional guidelines and in compliance with the Association for Research in Vision and Ophthalmology (ARVO) statement. The reporting of animal experiments was done in compliance with the ARRIVE guidelines. NDPK-B^−/−^ mice were generated as previously described [5]. Mice were sacrificed at 5 months of age and the retinas were isolated for immunoblotting or whole-mount immunofluorescence. Diabetes was induced in 2-month-old male mice using streptozotocin (STZ, i.p. 145 mg/kg body weight) (Roche, Mannheim, Germany) dissolved in citrate buffer (pH 4.5) as previously described [10]. Diabetic mice were sacrificed 3 months after diabetes induction for analysis of Tie2 expression.

### 4.3. Retinal Whole-Mount Immunofluorescence Staining

For whole-mount staining, the eyes were fixed in 4% paraformaldehyde (PFA) for 15 min followed by a wash with cold PBS for 5 min on ice. After dissection, the retinas were cut into four leaves at the periphery and fixed in methanol for 15 min. Then, the retinas were washed with PBS and incubated with permeabilization/blocking solution (2% BSA, 0.5% Triton X dissolved in PBS) for 1 h at room temperature. Post-incubation, the retinas were incubated with anti-Tie2 antibody (AF762, R&D Systems, Germany, 1:100) and anti-Lectin-TRITC (L5264, Sigma, Germany, 1:50) overnight at 4 °C. The retinas were then washed and incubated for 1 h at room temperature with the FITC-conjugated secondary antibody (R1254F, Acris, Germany, 1:200). Finally, the retinas were mounted on glass slides with mounting medium (HP19.1, Roth, Karlsruhe, Germany). Images were taken using confocal microscopy (Leica Microsystems, Germany).

### 4.4. Endothelial Immunofluorescence Staining

NDPK-B depleted and control HUVECs were seeded in 24 well plates on sterilized coverslips coated with 1% gelatin. After 24 h, serum starvation was performed with ECGM + 0.5% FCS for 24 h. The cells were further stimulated with high glucose (30 mM) in ECGM + 0.5% FCS for 24 h. On the day of staining, cells were washed with PBS and fixed with 4% formaldehyde. After permeabilization and blocking with 2.5% BSA and 0.3% Triton X-100 for 1 h, the cells were incubated with the primary antibody anti-Tie2 (Tie2 clone AB33, Merck Millipore, 1:100) overnight at 4 °C. Post-incubation, the cells were washed and incubated with the secondary antibody (Donkey anti-mouse Cy3, Jackson Labs 715-166-150, 1:200). Finally, the cells were mounted with Roti-Mount FluorCare (HP19.1, Roth, Karlsruhe, Germany). Photos were taken by confocal laser scanning microscopy (Leica Microsystems, Wetzlar, Germany). Quantification of the membrane protein expression in immunofluorescence was performed using Image J (NIH, Bethesda, MD, USA).

### 4.5. Isolation and Culture of Endothelial Cells

The use of Human Umbilical Vein Endothelial Cells (HUVECs) was approved (2012-388N-MA, 08/11/2018) by the local medical ethics committee (Medical Faculty Mannheim, Heidelberg University, Germany). HUVECs were isolated from the umbilical cords of newborn infants with the informed consent of their mothers. The isolation and culture of HUVECs have been previously illustrated [9]. HUVECs were harvested from the human umbilical vein via enzymatic dispase digestion and subsequently cultured in ECGM supplemented with growth factors (ECGM C-22010, PromoCell, Heidelberg, Germany) and 10% FCS (Sigma-Aldrich, Germany) on 1% gelatin-coated flasks. Cells up to passage 3 were used for experiments. HUVECs were used in the study unless otherwise stated. Human Retinal Microvascular Endothelial Cells (HRMVECs) (PB-CH-160-8511, Pelobiotech, Germany) and Human Microvascular Endothelial Cells (HMECs) (INS-CI-1008, inSCREENex, Germany) were cultured on 1% gelatin-coated flasks and passaged identical to HUVECs. HRMVECs were maintained in ECGM 10% FCS medium and HMECs were maintained in microvascular ECGM + 10% FCS (ECGM MV C-22020, PromoCell, Germany).

### 4.6. Cell Transfection

70% confluent HUVECs/HRMVECs/HMECs were transfected with the target siRNA using Lipofectamine RNAiMAX (13778030, Life Technologies, Darmstadt, Germany). Suitable scramble siRNA served as a control. NDPK-B-specific siRNA: AAC UGG UUG ACU ACA AGU CUU (Eurofins MWG, Ebersberg, Germany); Scrambled siRNA for NDPK-B: AGG UAG UGU AAU CGC CUU G (Eurofins MWG, Ebersberg, Germany); Tie2-specific siRNA and the respective control (SI00604912 and SI03650318, respectively Qiagen, Hilden, Germany). 4 h post-transfection, the cells were supplemented with ECGM in 10% FCS and maintained overnight in a humidified incubator at 37 °C and 5% CO2. Transfected cells were starved in ECGM + 0.5% FCS for 24 h. Treatment with sTie2 or HG was performed in growth factor-supplemented ECGM containing 0.5% FCS for 24 h.

### 4.7. Subcellular Fractionation

Fractionation of cells was performed with a commercially available kit (78840, ThermoFischer Scientific, Waltham, MA, USA). HUVECs were cultured and treated accordingly. Cells were trypsinized and subjected to cytoplasm, nuclear, and membrane isolation buffer as described in the manufacturer’s protocol. After separation, the respective fractions were dissolved in 4× Laemmli sample buffer and denatured at 95 °C for 5 min. The obtained fractions were used further for immunoblotting.

### 4.8. Immunoprecipitation

Cells seeded on 10 cm dishes were washed with ice-cold PBS and harvested by scraping in lysis buffer (50 mM Tris-HCl pH 7.4, 150 mM NaCl, 1% Triton X-100) containing cOmplete™ (protease inhibitor 1 tablet/10 mL buffer, Roche, Germany) and PhosStop™ (phosphatase inhibitor, 1 tablet/10 mL buffer Sigma Aldrich, Germany). The lysate was incubated on ice for 20–30 min and centrifuged at 13,000× *g* for 15 min at 4 °C. The supernatant was collected and incubated with the Tie2 precipitating antibody (AF313, R&D, UK, 2 µg per IP) on a top-down shaker at 4 °C for 2 h. 60 μL of the supernatant was set aside as Input control. For each sample, 40 μL of agarose beads were washed by lysis buffer. The beads were added to the lysate antibody mixture and incubated on a rotator at 4 °C overnight. After the incubation, the beads were collected by centrifugation and washed with lysis buffer containing PhosStop™. The immune complexes on the beads were then denatured with 1X Laemmli buffer, and the lysates were investigated by immunoblotting. Detection of Tie2 was performed with rabbit anti-Tie2 (Santa Cruz, Heidelberg, Germany, 1:500), and of phosphorylation with mouse-anti-pTyrosine 4G10 (05-321, Millipore, Darmstadt, Germany, 1:1000).

### 4.9. Immunoblotting

Immunoblotting was performed using proteins extracted from ECs and retinas in RIPA buffer (50 mM Tris-HCl, pH7.4, 150 mM NaCl, 1 mM dithiothreitol, 1% Triton X-100, 1% sodium deoxycholate) containing protease inhibitor (1 tablet/10 mL, Roche, Germany). The proteins were separated by SDS-PAGE and electrically transferred onto nitrocellulose membranes. After blocking with Roti-block (A151.1, Carl Roth, Karlsruhe, Germany, 1X solution in H_2_O), membranes were incubated with the respective primary antibodies overnight. Immune complexes were incubated with corresponding secondary antibodies and visualized using a chemiluminescent peroxidase substrate (34094, Thermo Scientific, Germany). Protein expression was quantified using ImageJ (NIH, USA).

### 4.10. ELISA

HUVECs were treated as illustrated in the results. The supernatants were collected and centrifuged to exclude cellular debris. These cleared supernatants were then used to detect secreted Ang2 using the human Angiopoietin2 DuoSet kit (DY623, R&D Systems, Minneapolis, MN, USA) according to the manufacturer′s instructions.

### 4.11. Adenovirus-Mediated Overexpression

HUVECs were transfected with NDPK-B siRNA, then starved in ECGM + 0.5% FCS for 24 h, followed by infection with Ad-Ang2 [31] in ECGM + 0.5% FCS for 24 h. ECs infected with Ad-GFP served as control. Infected cells were monitored for GFP expression. The cell supernatant was collected for confirmation of Ang2 overexpression using the Ang2 ELISA DuoSet kit. The pelleted cells were harvested analyzed for protein expression by immunoblotting.

### 4.12. Statistical Analysis

Data are represented as mean ± SD. Student’s *t*-test or Analysis of Variance (ANOVA) with Tukey post-test was performed using GraphPad Prism 6 (GraphPad Software, USA). *P* values < 0.05 were considered statistically significant.

## Abbreviations

NDPK-B	Nucleoside diphosphate kinase B
ECs	Endothelial cells
Ang2	Angiopoietin 2
HG	High glucose
NDPKs	Nucleoside diphosphate kinases
DR	Diabetic retinopathy
NVU	Neurovascular unit
PC	Pericytes
AC	Acellular capillaries
Ang1	Angiopoietin 1
VEGF	Vascular endothelial growth factor
sTie2	Soluble Tie2
HUVECs	Human umbilical vein endothelial cells
ECGM	Endothelial cell growth medium
HRMVECs	Human retinal microvascular endothelial cells
HMECs	Human microvascular endothelial cells
